# Upregulation of MircoRNA-370 Induces Proliferation in Human Prostate Cancer Cells by Downregulating the Transcription Factor FOXO1

**DOI:** 10.1371/journal.pone.0045825

**Published:** 2012-09-18

**Authors:** Ziqing Wu, Huabin Sun, Weixia Zeng, Jincan He, Xiangming Mao

**Affiliations:** 1 Department of Pathology, Nanfang Hospital, Southern Medical University, Guangzhou, P. R. China; 2 Department of Pathology, School of Basic Medical Sciences, Southern Medical University, Guangzhou, P. R. China; 3 Department of Urology, The Fifth Affiliated Hospital, Sun Yat-Sen University, Zhuhai, Guangdong Province, P. R. China; 4 Laura Biotech Co, Ltd., Guangzhou, Guangdong Province, P. R. China; 5 Department of Urology, Nanfang Hospital, Southern Medical University, Guangzhou, P. R. China; Northwestern University, United States of America

## Abstract

Forkhead box protein O1 (FOXO1), a key member of the FOXO family of transcription factors, acts as a tumor suppressor and has been associated with various key cellular functions, including cell growth, differentiation, apoptosis and angiogenesis. Therefore, it is puzzling why FOXO protein expression is downregulated in cancer cells. MicroRNAs, non-coding 20∼22 nucleotide single-stranded RNAs, result in translational repression or degradation and gene silencing of their target genes, and significantly contribute to the regulation of gene expression. In the current study, we report that miR-370 expression was significantly upregulated in five prostate cancer cell lines, compared to normal prostatic epithelial (PrEC) cells. Ectopic expression of miR-370 induced proliferation and increased the anchorage-independent growth and colony formation ability of DU145 and LNCaP prostate cancer cells, while inhibition of miR-370 reduced proliferation, anchorage-independent growth and colony formation ability. Furthermore, upregulation of miR-370 promoted the entry of DU145 and LNCaP prostate cancer cells into the G1/S cell cycle transition, which was associated with downregulation of the cyclin-dependent kinase (CDK) inhibitors, *p27^Kip1^* and *p21^Cip1^*, and upregulation of the cell-cycle regulator cyclin D1 mRNA. Additionally, we demonstrated that miR-370 can downregulate expression of FOXO1 by directly targeting the *FOXO1* 3′-untranslated region. Taken together, our results suggest that miR-370 plays an important role in the proliferation of human prostate cancer cells, by directly suppressing the tumor suppressor FOXO1.

## Introduction

Prostate cancer is the second common malignancy in males, with more than 0.78 million new cases occurring globally each year [1]. Approximately 190,000 men are diagnosed with prostate cancer and more than 27,360 prostate cancer patients die each year in the USA [2]. Prostate cancer represents a major public health concern and is associated with significant healthcare costs. Serum prostate-specific antigen (PSA) screening is useful for early diagnosis of prostate cancer; however, PSA screening has many shortcomings, for example, PSA levels are often elevated in men suffering benign prostate inflammation. The treatment strategies currently available for prostate cancer, including surgical castration and chemotherapy, are generally unsuccessful [3]. It is well known that prostate cancer lesions are heterogeneous and often respond well to initial androgen deprivation therapy (ADT) [4]. At present, most prostate cancer patients chose a gonadotropin-releasing hormone (GnRH) agonist/antagonist rather than surgical castration, and ADT is mainly applied as systemic therapy in patients with metastases. However, many prostate cancer patients eventually experience recurrence and androgen independence, which leads to accelerated disease progression and death [4], [5]. Hence, novel targets for effective prostate cancer treatment strategies urgently need to be identified.

Although both genetic and environmental factors are considered to be major factors, the molecular mechanisms of prostate cancer development and progression remain largely unknown. Malignant tumors are characterized by dysregulated activity in the regulatory pathways which control proliferation and/or apoptosis. The ability to inhibit one or more key targets within these signaling pathways may provide new breakthroughs in cancer treatment. Several regulatory pathways, such as the androgen receptor (AR) signaling pathway and Akt/protein kinase B (PKB) signaling pathway play a key role in the regulation of apoptosis and proliferation in prostate cancer cells 6–10. Hence, it is of importance to understand these pathways, as discovery of the key regulators may not only generate PrECise prognostic information, but may also provide novel treatment strategies for prostate cancer.

The Akt/PKB pathway promotes cell survival by regulating a number of transcription factors, including the forkhead transcription factor superfamily [11], such as Forkhead box protein O1 (FoxO1, also known as fork head in rhabdomyosarcomas [FKHR]), FoxO3a (FKHRL1), FoxO4 (AFX) and FoxO6. Since isolation of the forkhead gene in *Drosophila melanogaster*, more than 100 structurally-related forkhead transcription factors have been identified [12]. The members of the FOXO subfamily are evolutionarily conserved transcriptional activators, characterized by a highly conserved forkhead domain containing a DNA-binding motif [13]. FOXO1 was identified during study of the t(2,13)(q35;q14) and t(1,13)(p36;q14) chromosomal translocations, which are commonly found in alveolar rhabdomyosarcoma, a skeletal-muscle tumor prevalent in children [14]. FOXO proteins play a pivotal role in a variety of biological processes, including apoptosis, the cell cycle, differentiation, stress responses, DNA damage repair and glucose metabolism [15]. Activation of FOXO subfamily members can upregulate the cell-cycle inhibitors p21^Cip1^ and p27^Kip1^, and downregulate the cell cycle regulator cyclin D1/2, consequently leading to G1/S cell-cycle arrest [16]–[18]. Therefore, the FOXO transcription factors are key tumor suppressors. Growing evidence suggests that activation of FOXO1 induces apoptosis in prostate cancer cells [18]–[23]. Brunet et al. indicated that, by interacting with 14-3-3 chaperone proteins, phosphorylation may induce nuclear translocation of the FOXO transcription factors [13]. However, the reasons why FOXO is downregulated in cancer cells are poorly characterized. Thus, we hypothesized that an alternative mechanism may regulate FOXO protein expression in cancer cells.

MicroRNAs (miRNAs) are a class of small, non-coding, single-stranded RNAs which can negatively regulate gene expression at the post-transcriptional level, mainly by binding to the 3′ untranslated region (UTR) of their target mRNAs [24]–[26]. Numerous studies have demonstrated that aberrant expression of miRNAs is closely associated with proliferation, invasion, metastasis and the prognosis of various cancers, including prostate cancer, breast cancer, glioma and lung cancer [27]–[30]. Aberrant expression of miRNAs results in gene expression changes, epigenetic modifications and altered abundance of the miRNA-processing enzyme Dicer. Prostate cancer progression is associated with altered expression of multiple oncogenes and tumor suppressors, and miRNAs may potentially regulate these genes; however, the relationship between miRNAs and prostate cancer has only started to be elucidated in recent years [31]. More than 50 miRNAs are reported to be involved in prostate cancer; however, most of the current data suggests that only a small number of these relate to the pathogenesis of prostate cancer. Interestingly, of the miRNAs which are known to alter the phenotype of prostate cancer cells, some are considered oncogenic (e.g., miR-221/-222, miR-21 and miR-125b), while others are considered to be tumor suppressors (e.g., miR-101, miR-126*, miR-146a, miR-330, the miR-34 cluster and miR-200 family). Based on these findings, miRNAs are thought to have a number of potential clinical applications in prostate cancer as biomarkers and therapeutic targets [27], [30], [32]–[38].

In the current study, publicly available algorithms (TargetScan, Pictar, miRANDA) indicated that miR-370 may directly target the 3`-UTR of *FOXO1*. We demonstrated that miR-370 promoted prostate cancer cell proliferation by directly targeting the 3′-UTR of *FOXO1* mRNA, which subsequently reduced expression of the cyclin-dependent kinase (CDK) inhibitors, *p27^Kip1^* and *p21^Cip1^*, and upregulated the cell-cycle regulator *cyclin D1*. Our results suggest that miR-370 may play an important role in the development and progression of prostate cancer.

## Materials and Methods

### Cell culture

Normal prostate epithelial cells (PrEC) were obtained from Clonetics-Biowhittaker (Walkersville, MD, USA) and cultured in PrEMB medium (Clonetics-Biowhittaker). Prostate cancer cell lines Tsu-Pr1, PC3, DU145, 22Rv1 and LNCaP cell lines were obtained from the ATCC (Manassas, VA, USA). PC3 was maintained in F-12K Medium (Invitrogen), DU145 was cultured in Eagle's Minimum Essential Medium(Invitrogen), and Tsu-Pr1,22Rv1 and LNCaP were cultured in RPMI-1640 Medium(Invitrogen), supplemented with 10% fetal bovine serum (HyClone, Logan, UT, USA) and 1% penicillin/streptomycin (Invitrogen).

### Plasmids and transfection

The clone sequence of *FOXO1* 3′UTR is as following: CTTCAGATTGTCTGACAGCAGGAACTGAGAGAAGCAGTCCAAAGATGTCTTTCACCAACTCCCTTTTAGTTTTCTTGGTTAAAAAAAAAAACAAAAAAAAAAACCCTCCTTTTTTCCTTTCGTCAGACTTGGCAGCAAAGACATTTTTCCTGTACAGGATGTTTGCCCAATGTGTGCAGGTTATGTGCTGCTGTAGATAAGGACTGTGCCAT.

This sequence was amplified by PCR from PREC RNA and cloned into the *Sac*I/*Xma*I sites of the pGL3-control luciferase reporter plasmid (modified by adding *Sac*I/*Xma*I sites to plasmids from Promega, Madison, WI, USA) and the *Sac*I/*Xma*I sites of pGFP-C3 (modified by adding *Sac*I/*Xma*I sites to plasmids from Clontech, Mountain View, CA, USA). We have modified the original pGL3-control Vector by digesting the site of Xbal 1934 and destroying multiply cloning sites (MCS) which is in the upstream of the promoter SV40, so the original sites of *Sac*I/*Xma*I are lost. And then we have synthesized a section of sequence including *Sac*I/*Xma*I sites. Later we added this sequence to the site of Xbal 1934. The pGL3-control modified sequence is as following:

TCTAGA AA GAGCTC AACCAATGCATTGGTCC CCCGGG GGAGCTCTAGA

After all these, FOXO1 3′UTR is cloned into the 3′UTR of luc+ not the upstream of the promoter SV40.

In pGFP-C3 Plasmid, MCS is located in the 3′UTR of GFP, including the following sequence:

TACAAGTACTCAGATCTCGAGCTCAAGCTTCGAATTCTGCAGTCGACGGTACCGCGGGCCCGGGATCCACCGGATCTAGATAACTGATCA.

The primers were: *FOXO1*-3′UTR-wt-up: 5′-GCCCCGCGGCTTCAGATTGTCTGACAGCAGGAAC-3′; *FOXO1*-3′UTR-wt-dn:5′-GCCCTGCAGATGGCACAGTCCTTATCTACAGC-3′; *FOXO1*-3′UTR-mu-up: 5′-GCCCCGCGGCTTCAGATTGTCTGACAGCACCAAC-3′ and *FOXO1*-3′UTR-mu-dn:5′-GCC CTGCAGATGGCACAGTCCTTATCTACAGC-3′. The p3x IRS-MLP-luc plasmid was constructed as previously described [19]. The miR-370 mimics, negative control and anti-miR-370 inhibitor were purchased from RiboBio (Guangzhou, Guangdong, China). Transfection of the microRNA and microRNA inhibitor was performed using Lipofectamine 2000 (Invitrogen) according to the manufacturer's instructions.

### Western blotting

Western blot analysis was performed according to standard methods using anti-FOXO1, anti-p21, anti-p27, anti-cyclin D1, anti-Ki-67 antibodies (Cell Signaling, Danvers, MA, USA), anti-Rb and anti- phosphorylated Rb antibodies (Abcam, Cambridge, MA, USA). The membranes were stripped and re-blotted with an anti-ß-tubulin monoclonal antibody (Sigma, St. Louis, MO, USA) as a loading control.

### RNA extraction and real-time quantitative PCR

Total miRNAs were extracted from cultured cells using the mirVana miRNA Isolation Kit (Ambion, Austin, TX, USA) according to the manufacturer's instructions, then cDNA was synthesized from 5 ng of total RNA using the Taqman miRNA reverse transcription kit (Applied Biosystems, Foster City, CA, USA). The expression levels of miR-370 were quantified using a miRNA-specific TaqMan MiRNA Assay Kit (Applied Biosystems). The miRNA expression levels were defined based on the threshold cycle (C_t_), and the relative expression levels were calculated as 2^−[(Ct of miR-370) – (Ct of U6)]^.

Real-time PCR was performed using the Applied Biosystems 7500 Sequence Detection system using the following primers for *p21^Cip1^*,(forward, 5′-CGATGCCAACCTCCTCAACGA-3′ and reverse, 5′TCGCAGACCTCCAGCATCCA-3′); *p27^ Kip1^*(forward, 5′-TGCAACCGACGATTCTTCTACTCAA-3′ and reverse, 5′-CAAGCAGTGATGTATCTGATAAACAAGGA-3′) and cyclin D1 mRNA (forward, 5′-AACTACCTGGACCGCTTCCT-3′ and reverse, 5′-CCACTTGAG CTTGTTCACCA-3′). The expression data were normalized to the geometric mean expression level of the housekeeping gene *GAPDH* (forward, 5′-GACTCATGACCACAGTCCATGC-3′; reverse, 3′-AGAGGCAGGGATG ATGTTCTG -5′) and calculated using 2^−[(Ct of *p21, p27, or cyclin D1*) – (Ct^ ^of *GAPDH*)]^, where C_t_ represents the threshold cycle for each transcript.

### 3-(4, 5-Dimethyl-2-thiazolyl)-2, 5-diphenyl-2H-tetrazolium bromide (MTT) assay

Cells were seeded into 96-well plates. At the indicated time points, the cells were incubated with 100 μl sterile MTT (0.5 mg/ml, Sigma) for 4 h at 37°C, then the media was removed and replaced with 150 μl dimethyl sulphoxide (DMSO, Sigma). Absorbance was measured at 570 nm, with 655 nm as a reference wavelength. All experiments were performed in triplicate.

### Anchorage-independent growth ability assay

Five hundred cells were trypsinized and resuspended in 2 ml complete media containing 0.3% agar (Sigma). The agar–cell mixture was plated onto complete media containing 1% agar. After 10 days, the viable colonies were measured using an ocular micrometer and colonies containing more than 50 cells and colonies larger than 0.1 mm in diameter were counted. The experiment was performed three independent times for each cell line.

### Colony formation assays

Cells were plated in 6-well plates (0.5×10^3^ cells per plate), cultured for 10 days, fixed with 10% formaldehyde for 5 min, stained with 1.0% crystal violet for 30 s and counted.

### Bromodeoxyuridine labeling and immunofluorescence

Cells grown on coverslips (Fisher, Pittsburgh, PA, USA) were incubated with bromodeoxyuridine (BrdU) for 1 h then stained with an anti-BrdU antibody (Upstate, Temecula, CA, USA) according to the manufacturer's instructions. Gray level images were acquired using a laser scanning microscope (Axioskop 2 plus, Carl Zeiss Co. Ltd., Jena, Germany).

### Luciferase assays

Prostate cancer cells (3.5×10^4^) were seeded in triplicate in 24-well plates, allowed to settle for 24 h and then co-transfected with 100 ng p3x IRS-MLP luciferase plasmid DNA, 100 ng pGL3-FOXO1-3′UTR(wt/mu) plasmid DNA or 100 ng pGL3 control-luciferase plasmid and 1 ng of the control Renilla plasmid pRL-TK (Promega) using Lipofectamine 2000 (Invitrogen) according to the manufacturer′s instructions. Luciferase and Renilla activity were measured 48 h after transfection using the Dual Luciferase Reporter Assay Kit (Promega) according to the manufacturer′s instructions. Three independent experiments were performed and the data are presented as the mean ± SD. Luciferase activity values normalized to Renilla activity.

### Flow cytometry analysis

Cells were harvested by trypsinization, washed in ice-cold PBS, fixed in ice-cold 80% ethanol in PBS, centrifuged at 4°C and resuspended in chilled PBS. Bovine pancreatic RNAase (Sigma-Aldrich) was added at a final concentration of 2 μg/ml, incubated at 37°C for 30 min, then 20 μg/ml propidium iodide (Sigma-Aldrich) was added and incubated for 20 min at room temperature. In each group, 50,000 cells were analyzed by flow cytometry (FACSCalibur; BD Biosciences, San Jose, CA, USA).

### Statistical analysis

The two-tailed Student's *t*-test was used to evaluate the significance of the differences between two groups; *P* values <0.05 were considered significant.

## Results

### MiR-370 is upregulated in prostate cancer cell lines

**Figure 1:**
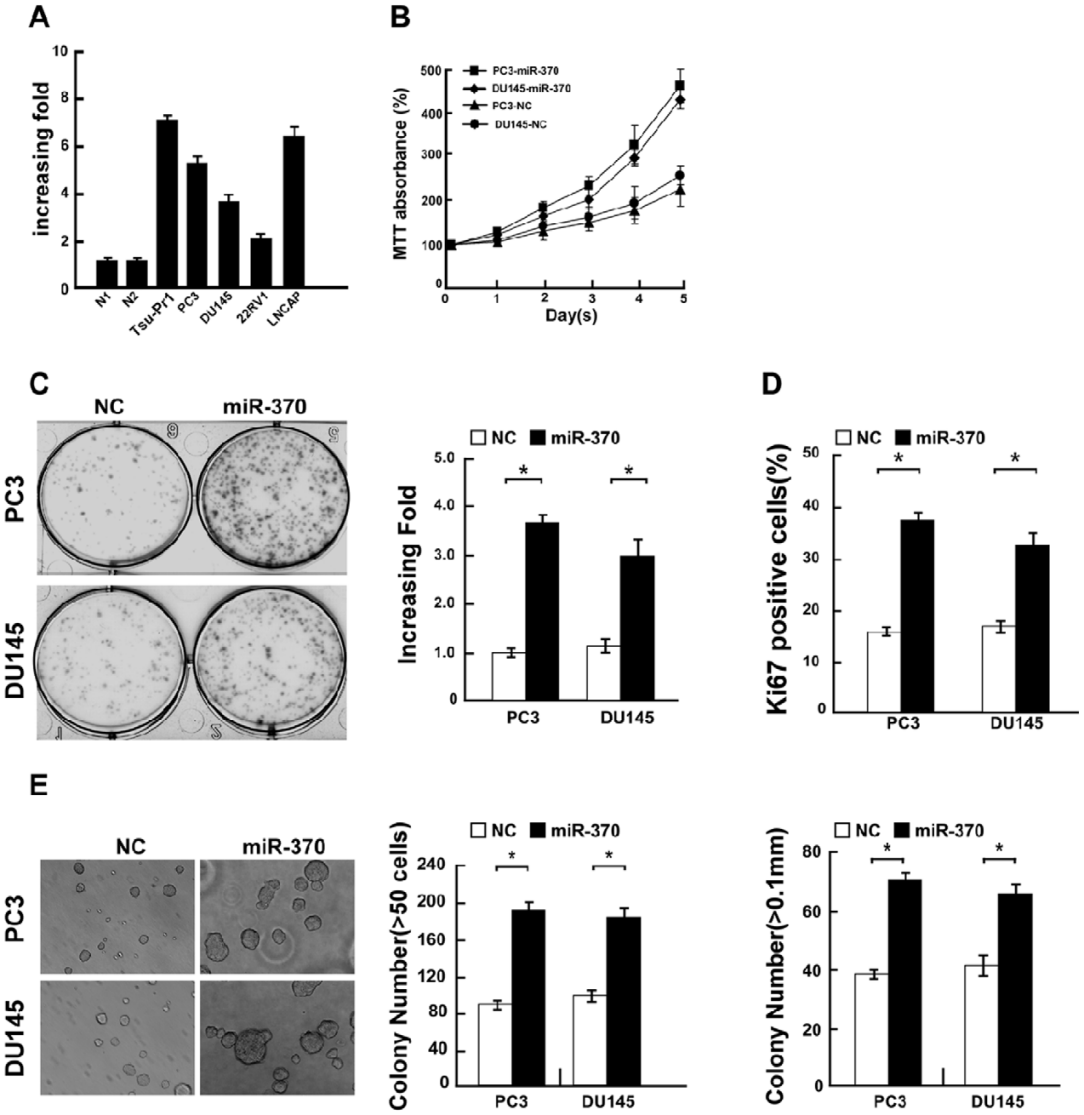
Upregulation of miR-370 promotes the proliferation of prostate cancer cells. **A**, Real-time PCR analysis of miR-370 expression in normal prostate epithelial cells (PrECs; shown as N1 and N2) and prostate cancer cell lines including PC3, DU145, 22Rv1 and LNCaP cells **B**, MTT assays indicating that the proliferation of miR-370-transfected cells increased, compared to negative control (NC)-transfected cells. **C,** Colony formation assay of miR-370 overexpressing cells; representative micrographs (left) and quantification (right) of crystal violet stained cell colonies. **D,** Quantification of Ki-67 positive cells in PC3 and DU145 cells transfected with miR-370 mimic or negative control (NC) 48 hours after transfection. **E,** Upregulation of miR-370 promoted prostate cancer cell tumorigenicity; representative micrographs (left) and quantification of colonies containing more than 50 cells (middle) or colonies larger than 0.1 mm (right) in the anchorage-independent growth assay. Bars represent the mean ± SD values of three independent experiments; *P<0.05.

Real-time PCR analysis revealed that miR-370 expression was markedly increased in all five prostate cancer cell lines tested (Tsu-Pr1, PC3, DU145, 22Rv1 and LNCaP), compared to normal prostate epithelial (PrEC ) cells (Figure 1A), indicating that miR-370 is upregulated in prostate cancer cell lines.

### Overexpression of miR-370 increases proliferation and enhances tumorigenicity

In order to investigate the function of miR-370 in prostate cancer, we transfected a hsa-miR-370 mimic into PC3 and DU145 prostate cancer cells and measured cell proliferation. Using MTT and colony formation assays, we observed that the growth rate of miR-370 overexpressing cells was dramatically increased, compared to negative control (NC)-transfected prostate cancer cells (Figure 1B and 1C). Furthermore, the proportion of Ki-67 positive cells, a known indicator of proliferating cells, was significantly increased in cells ectopically expressing miR-370, compared to NC-transfected cells (Figure 1D). These results demonstrated that upregulation of miR-370 promotes the proliferation of prostate cancer cells.

Moreover, ectopic expression of miR-370 in PC3 and DU145 prostate cancer cells significantly enhanced the anchorage-independent growth ability and lead to increased colony numbers and size in the soft agar colony formation assay (Figure 1F), suggesting that upregulation of miR-370 increased prostate cancer cell tumorigenicity *in vitro*. Taken together, these experiments demonstrated that upregulation of miR-370 promoted the proliferation and transformation of prostate cancer cells.

### Overexpression of miR-370 promotes the G1/S cell cycle transition in prostate cancer cells

**Figure 2:**
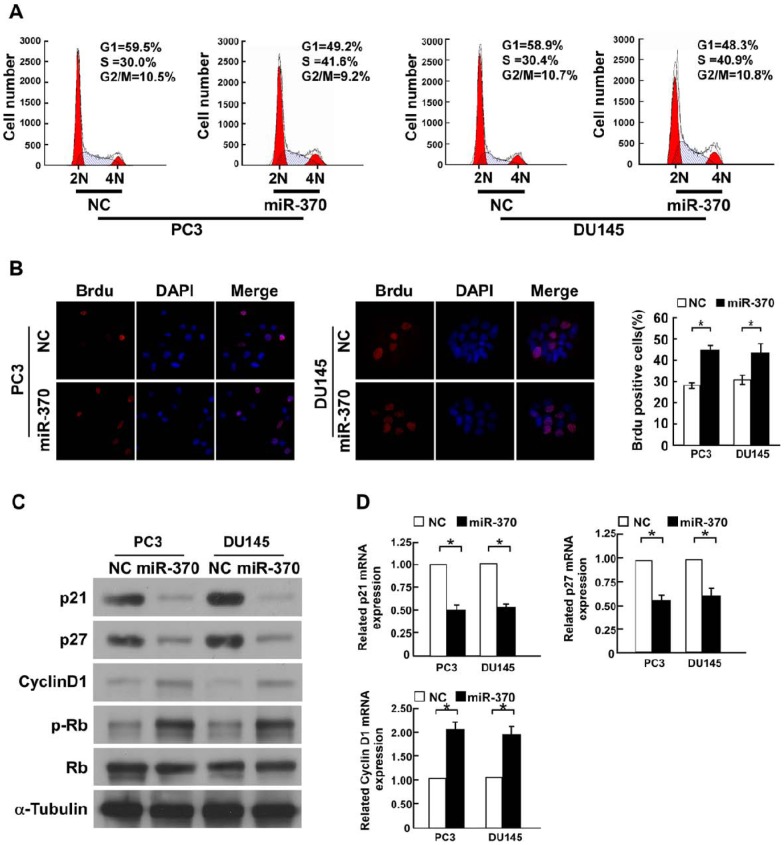
MiR-370 induces proliferation by promoting the G1-S transition. A , Flow cytometric analysis of PC3 and DU145 cells transfected with miR-370 mimic or negative control (NC) 48 hours after transfection. **B,** Representative micrographs (left) and quantification (right) of the BrdU incorporation assay in PC3 and DU145 cells transfected with miR-370 or NC 48 hours after transfection. **C,** Western blotting analysis indicating decreased expression of p21^Cip1^, p27^Kip1^ and increased expression of cyclin D1 and phosphorylated Rb (p-Rb) in PC3 and DU145 cells transfected with miR-370 or NC 48 hours after transfection. α-tubulin was used as a loading control. **D,** Real time PCR analysis of *p21^Cip1^, p27^Kip1^* and cyclin D1 mRNA in PC3 and DU145 cells transfected with miR-370 or NC 48 hours after transfection. *GAPDH* was used as a loading control. Bars represent the mean ± SD values of three independent experiments; *P<0.05.

We further investigated the effect of miR-370 on proliferation using flow cytometry. MiR-370-overexpressing PC3 and DU145 cells had a significantly lower percentage of cells in the G1/G0 phase and increased percentage of cells in the S phase, compared to NC-transfected cells (Figure 2A). Additionally, increased numbers of BrdU-incorporating cells were observed in miR-370-transfected cells, compared to NC-transfected cells (Figure 2B). Collectively, this data suggested that overexpression of miR-370 may enhance the proliferation of prostate cancer cells by promoting the G1/S cell cycle transition.

### MiR-370 decreases expression of the cell-cycle inhibitors *p21^Cip1^* and *p27^Kip1^* and increases expression of cell cycle regulator cyclin D1

As miR-370 promoted cell proliferation, we explored the effect of miR-370 on expression of the genes which regulate the G1/S transition [4]–[6], including the CDK inhibitors p21^Cip1^ and p27^Kip1^ and the CDK regulator cyclin D1. Using Western blotting and real-time PCR analysis, we observed that p21^Cip1^ and p27^Kip1^ protein and mRNA were downregulated and cyclin D1 protein and mRNA were upregulated in miR-370-transfected cells, compared to NC-transfected cells (Figure 2C and 2D). Coincident with altered expression of cell-cycle regulators, the phosphorylation level of Rb, a downstream target protein of CDK, was significantly increased in miR-370-transfected cells (Figure 2C), further confirming that miR-370 can influence the proliferation of prostate cancer cells.

### Inhibition of miR-370 reduces the proliferation of prostate cancer cells

**Figure 3:**
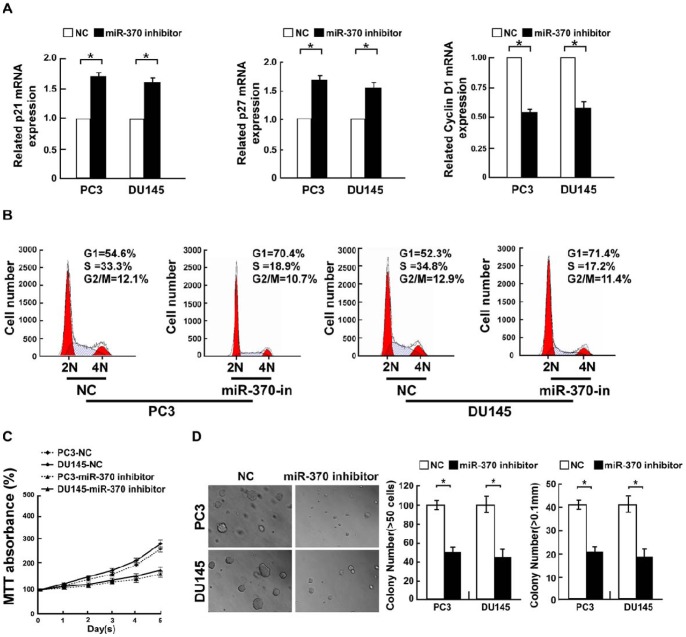
Inhibition of miR-370 suppresses the growth of prostate cancer cells. A, Real-time PCR analysis of *p21^Cip1^, p27^Kip1^* and cyclin D1 mRNA in PC3 and DU145 cells transfected with miR-370 inhibitor or negative control (NC) 48 hours after transfection. *GAPDH* was used as a loading control. **B,** Flow cytometric analysis of PC3 and DU145 cells transfected with miR-370 inhibitor or NC 48 hours after transfection. **C,** MTT assays revealed that inhibition of miR-370 reduced cell growth. **D,** Representative micrographs (left) and quantification of colonies containing more than 50 cells (middle) or colonies larger than 0.1 mm (right) in the anchorage-independent growth assays. Bars represent the mean ± SD values of three independent experiments; *P<0.05.

As described above, miR-370 plays a critical role in the proliferation of prostate cancer cells. However, it remained unknown whether inhibiting miR-370 would reduce cell proliferation. As expected, inhibition of miR-370 increased the transcription of *p21^Cip1^* and *p27^Kip1^* and reduced the expression of cyclin D1 mRNA (Figure 3A). Additionally, inhibition of miR-370 significantly increased the percentage of cells in the G0/G1 phase and decreased the percentage of cells in the S phase (Figure 3B). Moreover, using the MTT assay, we discovered that ectopic expression of the hsa-miR-370 inhibitor reduced the growth of PC3 and DU145 prostate cancer cells, compared to NC-transfected cells (Figure 3C). Additionally, as shown in Figure 3D, inhibition of miR-370 decreased the colony number and colony sizes of PC3 and DU145 cells in the colony formation assay, and also markedly reduced the anchorage-independent growth ability of both cell lines.

### MiR-370 directly targets the transcription factor *FOXO1* in prostate cancer cells

**Figure 4:**
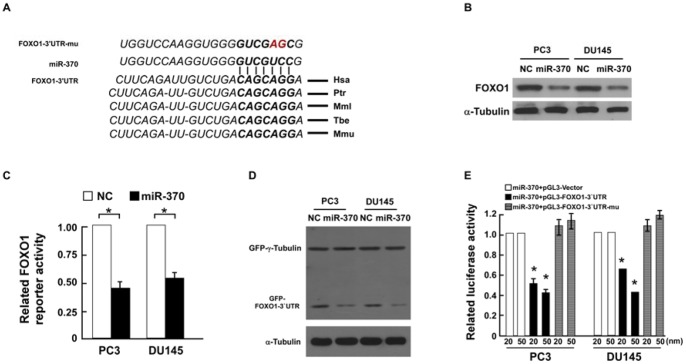
MiR-370 downregulates FOXO1 by directly targeting the *FOXO1* 3′UTR. **A**, Sequence of the *FOXO1* 3′UTR miR-370 binding seed region and mutation of the *FOXO1* 3′-UTR seed region to create FOXO1-mu. **B,** Western blotting analysis of FOXO1 expression in miR-370 or negative control (NC)-transfected PC3 and DU145 cells 48 hours after transfection. **C,** Relative FOXO1 reporter activities in miR-370 or NC-transfected cells 48 hours after transfection. **D,** Western blotting analysis of GFP reporter gene expression in miR-370 or NC-transfected cells 48 hours after transfection. **E,** Relative luciferase activity of PC3 or DU145 prostate cancer cells co-transfected with increasing amounts of miR-370 mimic oligonucleotides (20, 50 nM), and the pGL3 control reporter, pGL3-FOXO1-3′UTR reporter, or pGL3-FOXO1-3′UTR-mu reporter, 48 hours after transfection, respectively. Bars represent the mean ± SD values of three independent experiments; *P<0.05.

A previous study revealed that FOXO1 can regulate a series of genes relevant to the cell cycle at a transcriptional level, including *p21^Cip1^*, *p27^Kip1^* and cyclin D1 mRNA. In parallel, our analysis using three publicly available algorithms (TargetScan, Pictar, miRANDA) demonstrated that miR-370 may directly target the 3′-UTR of *FOXO1* (Figure 4A). This data indicated that miR-370 may modulate the expression of p27^ Kip1^, p21^Cip1^ and cyclin D1 by regulating *FOXO1*. As shown in Figure 4B, Figure S2A and Figure 4C, ectopic expression of miR-370 decreased the protein and mRNA expression levels of FOXO1 in PC3 and DU145 cells, indicating that *FOXO1* is a potential miR-370 target gene. FOXO1 is downregulated in prostate cancer cells (Figure S1A and Figure 1A); which is likely to be linked to upregulation of miR-370 in prostate cancer cells (Figure 1), which would reduce expression of the miR-370 target gene *FOXO1*.

To confirm the function of the putative miR-370 binding site in the *FOXO1* 3′-UTR, we cloned the *FOXO1* 3′-UTR into the reporter plasmids pEGFP-C3 and pGL3. GFP protein expression was dramatically inhibited by ectopic expression of miR-370 in PC3 and DU145 cells, compared to the control plasmid GFP-γ-tubulin, suggesting that miR-370 specifically targets the *FOXO1* 3′ UTR. Transfection of miR-370 consistently and dose-dependently reduced the luciferase activity of the *FOXO1* 3′-UTR luciferase reporter plasmid in PC3 and DU145 prostate cancer cells (Figure 4E). Furthermore, the repressive effect of miR-370 on the *FOXO1* 3′-UTR was abrogated by point mutations in the miR-370-binding seed region of the *FOXO1* 3′-UTR (Figure 4E). These results demonstrated that *FOXO1* is a *bona fide* target of miR-370.

**Figure 5:**
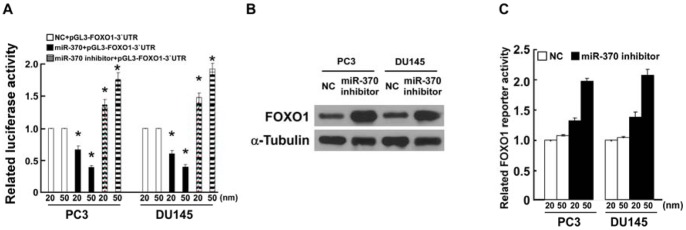
Inhibition of miR-370 activates the FOXO1 pathway. A, Relative luciferase activity assay of PC3 and DU145 cells co-transfected with the pGL3-FOXO1-3′UTR plasmid and increasing amounts (20, 50 nM) of miR-370 mimic- or miR-370 inhibitor-oligonucleotides, 48 hours after transfection, respectively. **B,** Western blotting analysis of FOXO1 expression in miR-370 or negative control (NC)-transfected PC3 and DU145 cells 48 hours after transfection. **C,** Luciferase activity assay of PC3 and DU145 cells transfected with the pGL3-FOXO1-3′UTR plasmid and increasing amounts (20, 50 nM) of miR-370 inhibitor-oligonucleotides 48 hours after transfection. Bars represent the mean ± SD values of three independent experiments; *P<0.05.

Transfection of a miR-370 inhibitor restored the luciferase activity of the pGL3-FOXO1-3′UTR reporter plasmid in PC3 and DU145 prostate cancer cells (Figure 5A), and upregulated FOXO1 protein expression (Figure 5B). Furthermore, inhibition of miR-370 consistently and dose-dependently increased the luciferase activity of pGL3-FOXO1-3′UTR in both prostate cancer cell lines (Figure 5C). Taken together, these results indicate that inhibition of miR-370 upregulated FOXO1.

To confirm the effect of miR-370 overexpression in prostate cancer cells (Figure 1), we quantified the expression of FOXO1 in prostate cancer cells. We observed that FOXO1 is downregulated in prostate cancer cells (Figure S1A and Figure 1A); confirming that overexpression of miR-370 downregulates the miR-370 target gene *FOXO1* in prostate cancer cells.

### MiR-370-induced prostate cancer cell proliferation is modulated by FOXO1

**Figure 6:**
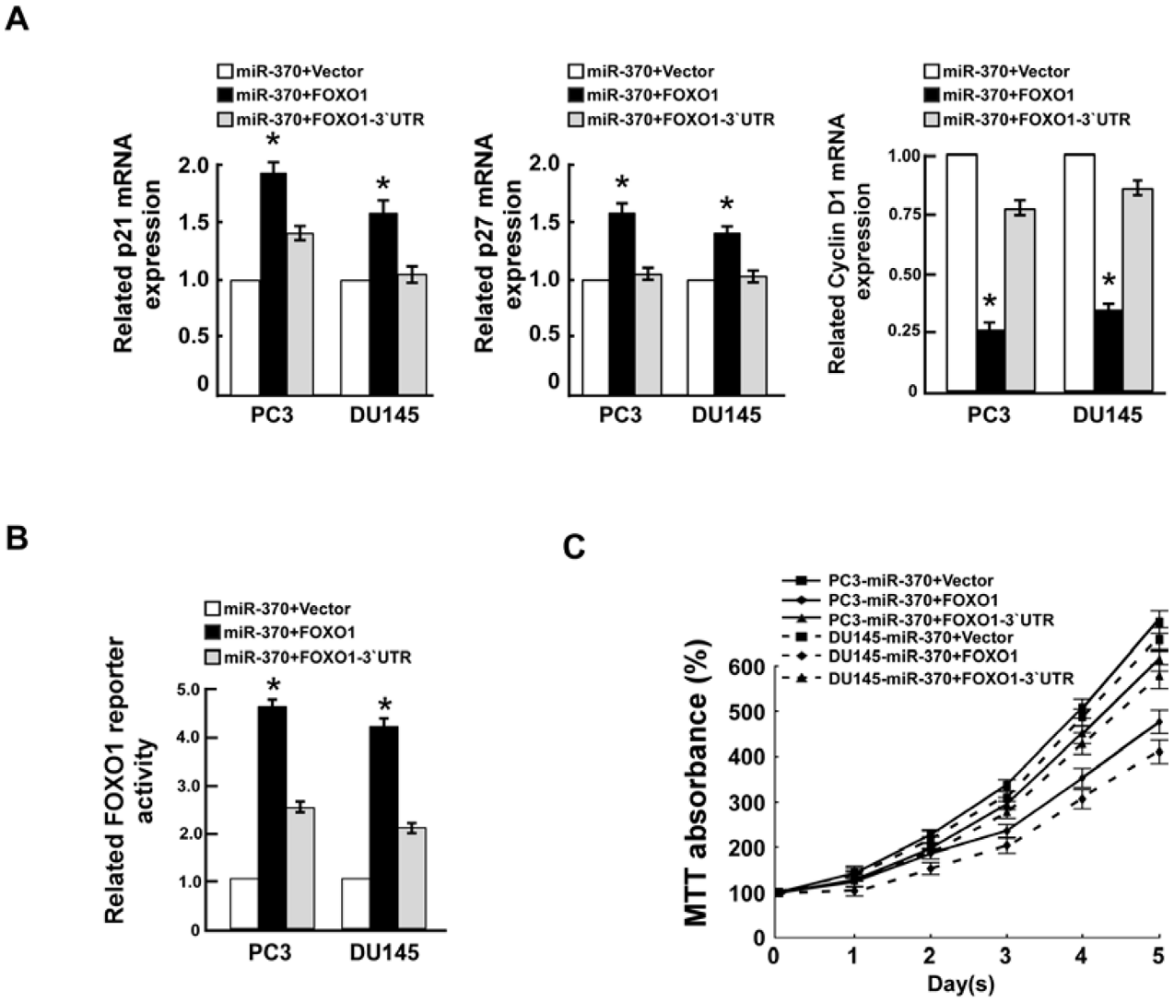
MiR-370-induced prostate cancer cell proliferation is mediated by FOXO1. A, Real-time PCR analysis of *p21^Cip1^, p27^Kip1^* and cyclin D1 mRNA expression in the indicated cells. *GAPDH* was used as a loading control 48 hours after transfection. **B,** Luciferase activity assay of the indicated cells transfected with a FOXO1 reporter 48 hours after transfection. **C,** MTT assay of prostate cancer cells transfected with miR-370 mimic, co-transfected with miR-370 and FOXO1, or co-transfected with miR-370 and FOXO1-3′-UTR.

To investigate whether FOXO1 could repress miR-370-induced proliferation, *FOXO1* (without the 3′-UTR) and *FOXO1*-3′-UTR (with the 3′-UTR) were transfected into miR-370-overexpressing prostate cancer cells. As expected, ectopic expression of FOXO1 lead to significantly greater changes in expression of *p27^ Kip1^*, *p21^Cip1^* and cyclin D1 mRNA than FOXO1-3′UTR (Figure 6A). In particular, after transfection of a FOXO1 reporter gene and miR-370 into PC3 and DU145 prostate cancer cells, luciferase activity could be restored by overexpression of FOXO1 and partially rescued by transfection of the FOXO1-3′-UTR (Figure 6B). Furthermore, the growth rate of both PC3 and DU145 prostate cancer cells was inhibited to a significantly greater extent by co-transfection of miR-370 and FOXO1 than co-transfection of FOXO1-3′-UTR and miR-370 (Figure 6C). In conclusion, these results suggest that miR-370-induced prostate cancer cell proliferation is directly mediated by suppression of *FOXO1*.

## Discussion

In this study, we demonstrated that miR-370 is upregulated in prostate cancer cell lines, compared to primary normal prostate epithelial cells. Upregulation of miR-370 promoted the G1/S cell cycle transition in prostate cancer cells, which correlated with downregulation of the cyclin-dependent kinase (CDK) inhibitors p27^Kip1^ and p21^Cip1^. Conversely, inhibition of miR-370 reduced prostate cancer cell proliferation, upregulated p27^Kip1^ and p21^Cip1^, and delayed the G1/S transition. MiR-370 upregulated the cell-cycle regulator cyclin D1 by directly targeting the *FOXO1* 3′-UTR, demonstrating that FOXO1 is regulated by miR-370 in prostate cancer cells. Collectively, these findings suggest that upregulation of miR-370 may promote the initiation and progression of prostate cancer.

It has been demonstrated that FOXO1 expression is regulated by several microRNAs, such as miR-223, miR-182, miR-27a, miR-139 and miR-96 [39]–[43]. Of these, some microRNAs may be dysregulated in prostate cancer, such as miR-27a, miR-182 [41], [44]. In fact, a single mRNA frequently could be regulated by multiple microRNAs in the regulation of genes. This could be because of the same seed sequence of microRNA or the different sites of target sequences in the 3′untranslated region of mRNA [43]. However there was no report about FOXO1 regulated by microRNA in prostate cancer to promote proliferation clearly and directly yet. It is the first for us to uncover the new relationship of regulation of FOXO1 and microRNA in prostate cancer. We observed that miR-370 was significantly overexpressed in prostate cancer cell lines, compared to PREC. Furthermore, ectopic overexpression of miR-370 significantly increased the growth of PC3 and DU145 cells, while suppression of miR-370 slowed proliferation and reduced colony-forming ability. We identified the tumor suppressor gene *FOXO1* as a putative miR-370 target gene using bioinformatic analysis, and confirmed that *FOXO1* is a *bona fide* target of miR-370. Ectopic overexpression of FOXO1 (without the 3′UTR) significantly abrogated miR-370-induced proliferation, whereas transfection of the FOXO1 3′UTR (containing the 3′UTR) only partially reduced miR-370-induced proliferation, suggesting that miR-370 increases the proliferation of prostate cancer cells by directly targeting the *FOXO1* 3′-UTR to downregulate FOXO1. Several other miRNAs have been identified to regulate the FOXO transcription factor family. For example, miR-27a, miR-96 and miR-182 can coordinately regulate the expression of FOXO1 by directly targeting the *FOXO1* 3′-UTR in breast cancer [34]. MiR-96 can regulate FOXO3a [35] and upregulation of miR-96 induces the proliferation of human breast cancer cells by downregulating FOXO3a [45].

In this study, overexpression of miR-370 increased cell proliferation and colony formation ability and decreased FOXO1 protein expression, which correlated with reduced expression of the genes regulated by FOXO1, including the cell-cycle inhibitors *p21^Cip1^* and *p27^Kip1^*, and upregulation of the cell-cycle regulator cyclin D1. In agreement with our results, downregulation of FOXO1 in chicken embryo fibroblasts or inhibition of the transcriptional activity of FOXO3a in human breast cancer cells can promote transformation and tumor progression [46], [47]. In contrast, ectopic expression of FOXO1 induces apoptosis in certain cancer cells, including prostate cancer cells. Additionally, it has been demonstrated that FOXO1-induces G1 phase cell-cycle arrest in renal cell carcinoma and glioma cells due to inhibition of tumor suppressor phosphatase and tensin homolog deleted on chromosome ten (PTEN), via upregulation of the cyclin-dependent kinase inhibitor p27^Kip1^ [20]. Moreover, FOXO-induced G1 arrest has been linked to downregulation of cyclin D1 and D2 [48].

FOXO1 has been characterized as a key tumor suppressor in prostate cancer. FOXO1 is involved in prostate cancer cell migration and invasion as a critical negative regulator of Runx2 [49]. Moreover, FOXO1 is a critical downstream effector of PTEN-mediated inhibition of AR activation, as FOXO1 inhibits androgenic activation of the AR and also abolishes androgen-independent AR activation [50]. CDK1 and CDK2, two cell cycle regulatory protein kinases important for the G1 to S and G2 to M cell cycle transitions, respectively, interact to phosphorylate FOXO1 at Ser 249 (S249) in prostate cancer cells [51], [52]. Indeed, treatment of prostate cancer cells with the CDK inhibitor roscovitine and the PI3K inhibitor LY294002 synergistically induces expression of the FOXO1 target gene BIM [53]. Phosphorylation of FOXO1 attenuates the tumor suppressor function of FOXO1, and induces prostate cancer cell growth and survival. Conversely, expression of FOXO1 restores the tumor suppressor function of FOXO1 and reduces prostate cancer cell growth and survival [54]. These observations indicate that activation of FOXO1 by inhibition of miR-370 may be a potential therapeutic strategy for prostate cancer.

A recent study demonstrated that hemizygous and homozygous deletions within the *FOXO1* gene locus are present in approximately 30% of prostate cancer cell lines, xenografts and a cohort of human prostate cancers [15]. The tumor suppressor function of FOXO1 can also be inhibited by protein kinase pathways [55]. Thus, the function of FOXO1 is frequently abolished via various mechanisms in human prostate cancer, further confirming the role of FOXO1 as a tumor suppressor. This study suggests that upregulation of miR-370 may provide an alternative mechanism for the reduced expression of the FOXO1 tumor suppressor protein in prostate cancer cells.

Further research is still required to examine whether other miRNAs or signaling pathways can regulate FOXO1 in prostate cancer, because we could not exclude that there might be other microRNAs, not found yet, to play an important role in regulating FOXO1 in prostate cancer, and whether miR-370 can target other members of the *FOXO* family. For example, it would be of interest to know whether other pathways are involved in the anti-proliferative effect of FOXO1 and investigate what other components of the malignant phenotype are determined by FOXO1 and miRNAs in prostate cancer cells, and these issues are currently under further investigation in our laboratory. A number of prostate-specific miRNAs are androgen-dependent, which may contribute to the complicated interactions of miRNAs and genes in prostate cancer. Regulation of miRNAs via androgen signaling-dependent mechanisms may possibly play a role in the transition to androgen-independent prostate cancer [27]. Further research is required to fully characterize the effect of the AR on the role of miR-370 in prostate cancer progression, and the role of miR-370 in the development of androgen independence.

In summary, the key finding of the current study is that miR-370 can increase the proliferation of prostate cancer cell lines by targeting *FOXO1*. This data indicates that miR-370 plays an essential role in the regulation of prostate cancer cell proliferation and may function as an onco-miRNA. Additionally, the upregulation of miR-370 may correlate with clinical progression in prostate cancer. Understanding the PrECise role played by miR-370 in prostate cancer progression will not only advance our knowledge of prostate cancer biology, but also will help determine if miR-370 has potential as a novel therapeutic target for the treatment of prostate cancer.

## Supporting Information

Figure S1 **FOXO1 was repressed in prostate cancer cell lines. A.** Western blotting analysis of FOXO1 expression in normal prostate epithelial cells (PrECs) and other prostate cancer cell lines.
(TIF)

Figure S2 **Targeting effect of miR-370 on FOXO1 in prostate cancer cells on day 10. A.** Western blotting analysis of FOXO1 expression of PC3 and DU145 cells transfected with miR-370 mimic or negative control (NC) on day 10.
(TIF)
