# Supplementary figures and images for: Upregulation of MircoRNA-370 Induces Proliferation in Human Prostate Cancer Cells by Downregulating the Transcription Factor FOXO1
Source: PLoS One. 2012 Sep 18;7(9):e45825. doi: 10.1371/journal.pone.0045825 (PMC3445500; doi:10.1371/journal.pone.0045825)

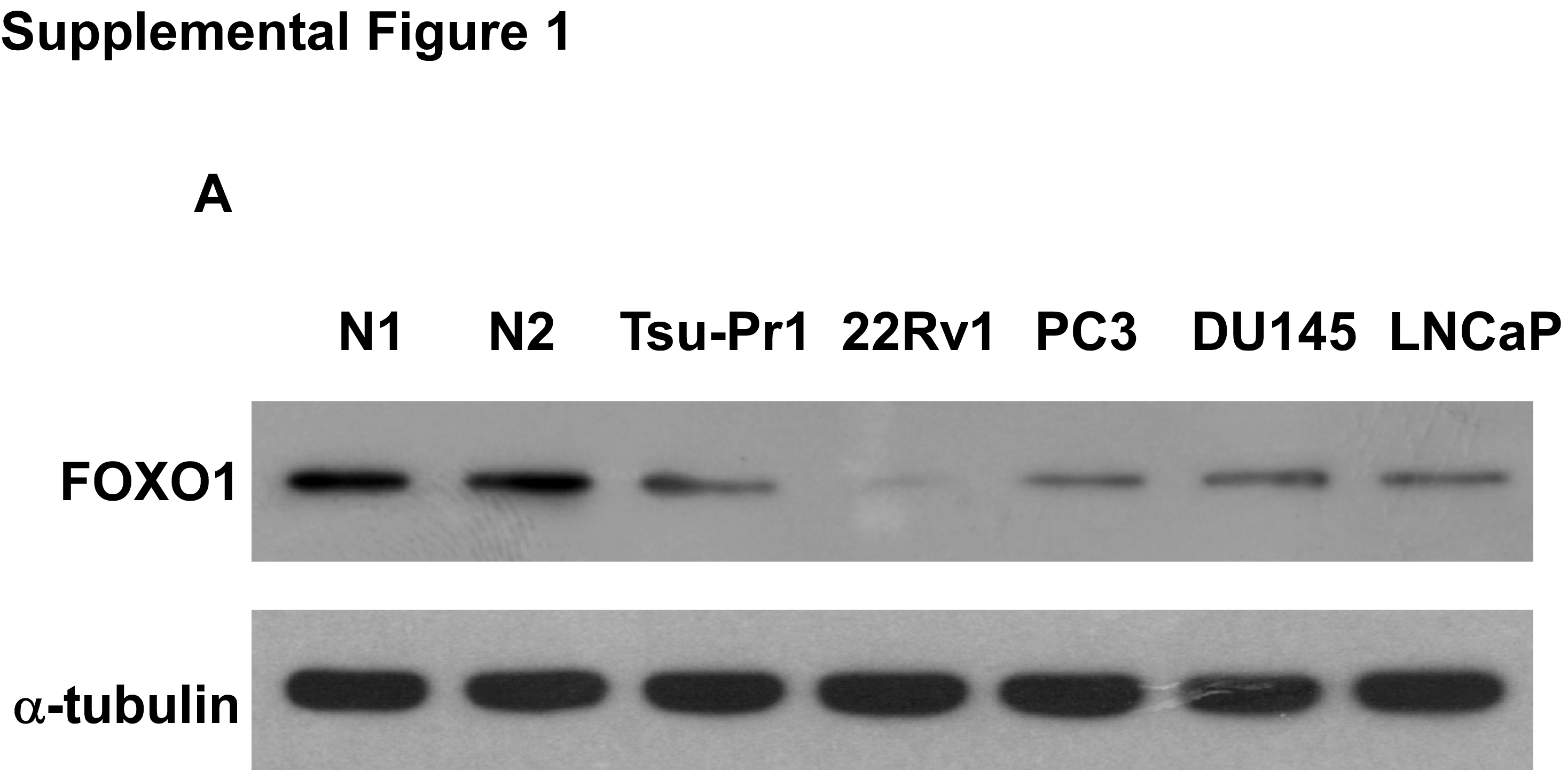

Supplement: Figure S1 — FOXO1 was repressed in prostate cancer cell lines. A. Western blotting analysis of FOXO1 expression in normal prostate epithelial cells (PrECs) and other prostate cancer cell lines. (TIF) [file pone.0045825.s001.tif]

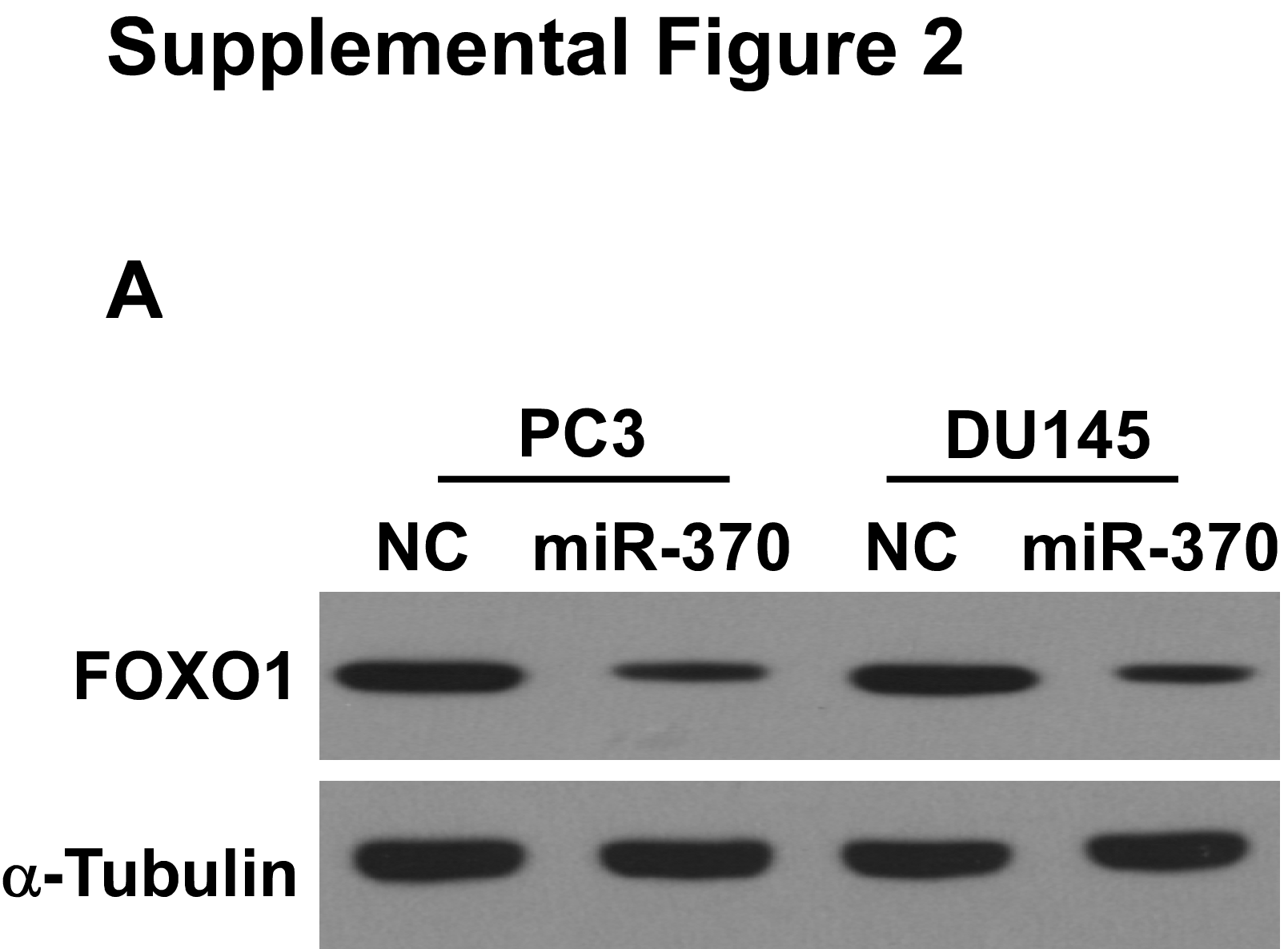

Supplement: Figure S2 — Targeting effect of miR-370 on FOXO1 in prostate cancer cells on day 10. A. Western blotting analysis of FOXO1 expression of PC3 and DU145 cells transfected with miR-370 mimic or negative control (NC) on day 10. (TIF) [file pone.0045825.s002.tif]
